# Early recovery of the platelet count after decitabine-based induction chemotherapy is a prognostic marker of superior response in elderly patients with newly diagnosed acute myeloid leukaemia

**DOI:** 10.1186/s12885-018-5160-5

**Published:** 2018-12-19

**Authors:** Jiayu Huang, Huihui Zhao, Ming Hong, Han Zhu, Yu Zhu, Yun Lian, Shan Li, Jianyong Li, Sixuan Qian

**Affiliations:** 1 Department of Hematology, The First Affiliated Hospital of Nanjing Medical University, Jiangsu Province Hospital, 300 Guangzhou Road, Nanjing, 210029 China; 2 Department of Oncology, The Second Affiliated Hospital of Southeast University, Zhongfu Road 1-1, Nanjing, 210003 China

**Keywords:** Platelet recovery, Prognostic indicator, Decitabine, Acute myeloid leukaemia, Elderly

## Abstract

**Background:**

Definite prognostic clinical factors of benefit for decitabine-based induction chemotherapy in elderly patients newly diagnosed with acute myeloid leukaemia (AML) are not identified. This study was designed to explore the potential biomarker, especially regeneration of haematopoiesis, of treatment response and survival in elderly patients with newly diagnosed AML.

**Method:**

We analysed the clinical data of 117 elderly AML patients who were treated with a decitabine dose of 15 mg/m^2^ for 5 days, granulocyte colony-stimulating factor of 300 μg/d for priming, plus cytarabine 10 mg/m^2^ q12h for 7 days and aclarubicin 10 mg/d for 4 days (D-CAG).

**Results:**

After initial induction chemotherapy, the overall response rate and complete remission (CR) were 71.8% and 58.1%, respectively. Patients responding to the D-CAG regimen achieved higher platelet counts on day 14 after initial treatment (*p* < 0.001). Median counts were 59.5 × 10^9^/L in the CR group, 37 × 10^9^/L in the partial remission group and 28 × 10^9^/L in the non-responsive group. We then classified patients into those who achieved platelet counts≥60 × 10^9^/L or 100 × 10^9^/L on day 14 after D-CAG vs. those who did not. Platelet counts≥60 × 10^9^/L or 100 × 10^9^/L on day 14 were significantly associated with superior CR, overall survival and disease-free survival (80.9% vs. 45.3% *p* < 0.001,16.5 vs. 9.1 months *p* = 0.009 and 16.3 vs. 7.4 months *p* = 0.024; 85.2% vs. 50% *p* = 0.001, 31 vs. 10.1 months *p* = 0.003 and 16.9 vs. 8.9 months *p* = 0.006). Multivariate analysis confirmed that poor cytogenetics (*p* = 0.010) and FLT3-ITD mutation (*p* = 0.007) were identified as independent factors of OS, but not platelet count (*p* = 0.091). However, platelet count≥100 × 10^9^/L on day 14 was an independent prognostic factor of CR and DFS.

**Conclusion:**

Platelet count recovery on day 14 after D-CAG induction chemotherapy is associated with response.

**Trial registration:**

D-CAG regimen was registered on ChicTR with number 11001700.

**Electronic supplementary material:**

The online version of this article (10.1186/s12885-018-5160-5) contains supplementary material, which is available to authorized users.

## Background

Acute myeloid leukaemia (AML) develops from clonal malignant disorders of haematopoietic stem cells, with a median age at diagnosis of 68 years [1]. Because of clinically and biologically heterogeneous features, elderly AML patients have poor prognosis attributable to increased resistance to current standard cytotoxic agents and/or poor tolerance of intensive induction chemotherapy; the rate of treatment-related mortality rate is 25–40% [1, 2]. Currently, decitabine is the appealing approach for AML patients who are not applicable for intensive induction chemotherapy. In a phase III study enrolling elderly patients (≥60 years) with previously untreated AML, decitabine resulted in a response rate (complete remission (CR) plus the CR without platelet recovery) of 17.8% and a median overall survival (OS) of 7.7 months [3]. However, this regimen was associated with low survival rates. The combination of decitabine and cytotoxic agents has been well established to trigger superior outcome and good tolerance for older or relapsed patients with AML [4, 5]. Our previous published study showed the regimen of low-dose decitabine, aclarubicin, cytarabine in combination with granulocyte stimulating factor (D-CAG) led to better results for elderly AML patients, with CR of 64.7% and median survival of 10 months [4]. Epigenetic changes are implicated in the mechanism of AML development and the hypomethylating agents received the US Food and Drug Administration approval for treatment in elderly AML patients. Methylation studies have shown global- and gene-specific hypermethylation in MDS and AML, but there seems to be little relation between the degree of demethylation following hypomethylating treatment and haematological response [6–8]. Effective methods for identifying AML patients who are most likely to respond to treatment with decitabine would be of immediate clinical utility [9].

In clinical practice, specific chromosome abnormalities and molecular genetic changes are of particular interest in term of prognosis, thus may be used for stratifying patients with AML at different risks, which contributes for identifying appropriate therapeutic strategies [10]. However, their ability to predict response or survival on decitabine therapy is not be distinctly elucidated [11]. Recently, low-dose decitabine has been shown to promote megakaryocyte maturation and platelet production [12, 13]. Platelet response was identified as an independent positive predictor of overall response and OS [14–16]. To our knowledge, the prognostic importance of platelet counts before haematological recovery for response to decitabine alone or decitabine-based chemotherapy, such as D-CAG, remains unknown. Therefore, it is hoped that early recovery in the platelet count before haematological recovery will allow us to optimize treatment of distinct subtypes of AML receiving decitabine-based chemotherapy.

In the analysis of this study, we analysed baseline biomarkers, molecular mutation, clinical variables, and regeneration of haematopoiesis after a first cycle of D-CAG chemotherapy to investigate a prognostic marker of CR and OS in elderly AML patients.

## Patients and methods

### Patients

One hundred and seventeen patients aged≥60 years with newly diagnosed de novo or *secondary* AML (Additional file 1) according to the International Working Group criteria was enrolled in this study [17]. We compiled the routine blood values of the patients receiving D-CAG on day 7, 10 and 14 after chemotherapy. The study procedures and informed consent forms were approved by the ethic committee of the First Affiliated Hospital of Nanjing Medical University, Jiangsu Province Hospital with number 2011-SR-085 and also registered on ChicTR with number 11001700. All patients or their legal trustee provided written informed consent.

### Treatment

All patients were administered decitabine at a dose of 15 mg/m^2^ intravenously (day 1–5) and granulocyte colony-stimulating factor of 300 μg/d (day 0–9) for priming in combination with cytarabine 10 mg/m^2^ q12h (day 3–9) and aclarubicin 10 mg/d (day 3–6) (D-CAG) as induction therapy. Hydroxyurea was permitted as rescue medication to control white blood cells (WBC) to< 5.0 × 10^9^/L but was discontinued at least 24 h before decitabine treatment. Red cells and platelets were infused if haemoglobin (Hb) was under 70 g/L or platelet count under 20 × 10^9^/L. Patients who did not achieve CR or partial remission (PR) were offered alternative therapies. Post-remission therapy consisted of 4–6 cycles D-CAG or conventional chemotherapy [4].

### Study assessments

Bone marrow aspiration was performed when peripheral hemogram recovered, or 3–4 weeks after chemotherapy. Cytogenetic risk groups and treatment response were determined by European Leukaemia Net [18] and International Working Group criteria [17]. Mutation analysis of four relevant molecular marker genes was carried out as described previously [4].

To quantify objective responses, CR was defined as normalization of bone marrow blasts (≤5% blasts) and peripheral blood neutrophil count ≥1.0 × 10^9^/L, platelet count > 100 × 10^9^/L. PR was defined as morphologic CR and 5–15% blasts with a decrease of at least 50% of total bone marrow blasts. The overall response rate (ORR) incorporated rates of CR and PR. All other patients were considered non-responders.

OS was measured from day 14 after the first cycle chemotherapy to the date of death from any causes or last follow-up. Disease-free survival (DFS) was calculated from the date of achievement of CR to an event, including relapse, death or last follow-up.

### Statistical analysis

Differences to response treatment efficacy in subgroups according to platelet count were evaluated using the rank sum test for non-normal data. Patient characteristics were compared using T test (counting variables), Chi-square test or Fisher’s exact test (categorical variables) between patients who did or did not achieve platelet count≥60 × 10^9^/L or 100 × 10^9^/L. The Chi-square test was also adopted for analysis of remission rate difference. A step multivariable logistic regression model was conducted for CR and ORR, as well as included covariates significant on univariate analysis. Kaplan-Meier method was performed to estimate the median survival and log-rank test was used to compare survival curves. To assess the independent prognostic variable on OS, hazard ratios (HR) and 95% confidence interval (CI) were calculated by using a Cox proportional hazards model. The covariates included ECOG PS, cytogenetic risk, FLT3-ITD and platelet count≥100 × 10^9^/L. A *P* value< 0.05 was considered statistically significant. All statistical analyses were performed by using SPSS Version 20 software.

## Results

### Patient characteristics

From September 2011 to April 2016, 117 newly diagnosed elderly AML patients were included in the study. The median age at diagnosis was 67 years (range: 60 to 87 years) with a male/female ratio of 1.21:1. Patients diagnosed with acute promyelocytic leukaemia were excluded from this study. Among those cases, 36 (30.8%) patients were aged 70 to 79, and 9 (7.7%) patients were aged 80 years or older. Baseline clinical characteristics for all patients are shown in Table 1.

**Table 1:** Baseline characteristics of the 117 patients with acute myeloid leukaemia

Characteristics	Total(*N* = 117)		
	N	Median(range)	%
Age		67(60–87)	
Sex			
Male	64		54.7
Female	53		45.3
ECOG			
0–2	96		82.1
≥ 3	21		17.9
Diagnosis			
M0	3		2.6
M1	19		16.2
M2	57		48.7
M4	8		6.8
M5	18		15.4
M6	10		8.6
Unknown	2		1.7
Primary karyotype			
Good	1		0.8
Intermediate	89		76.1
Poor	18		15.4
Unavailable	9		7.7
FLT3-ITD mutation			
Positive	13		11.1
Negative	88		75.2
Missing	16		13.7
NPM1 mutation			
Positive	24		20.5
Negative	78		66.7
Missing	15		12.8
CEBPα mutation			
Positive	16		13.7
Negative	81		69.2
Missing	20		17.1
C-Kit mutation			
Positive	4		3.5
Negative	94		80.3
Missing	19		16.2
Initial CBC			
WBC(×10^9^/L)		6.92(0.3–310.3)	
Hb(×g/L)		84(47–139)	
PLT(×10^9^/L)		49(8–804)	

*ECOG* Eastern Cooperative Oncology Group, *WBC* white blood cells, *Hb* hemoglobin, *PLT* platelet

Pre-treatment cytogenetics were determined in 108 (92.3%) patients: 1 (0.8%) had good cytogenetics, 18 (15.4%) had poor cytogenetics, and the remaining 89 (76.1%) had intermediate cytogenetics.

At the time of diagnosis of AML, the patients’ median WBC count was 6.92 (0.3–310.3) × 10^9^/L, the median Hb level was 84 (47–139) g/L, and the median platelet count was 49 (8–804) × 10^9^/L.

### Treatment response

All patients were eligible for response following the administration of D-CAG as induction therapy. A median number of 2 cycles of the D-CAG regimen (range from 1 to 7 cycles) were administrated. After the first cycle, ORR was 71.8% (84 patients); 68 (58.1%) patients achieved CR, and 16 (13.7%) patients had PR. After the second cycle, ORR and CR were 72.7% and 64.1%, respectively.

### Prognostic factors for treatment response in elderly patients with AML

**Fig. 1:**
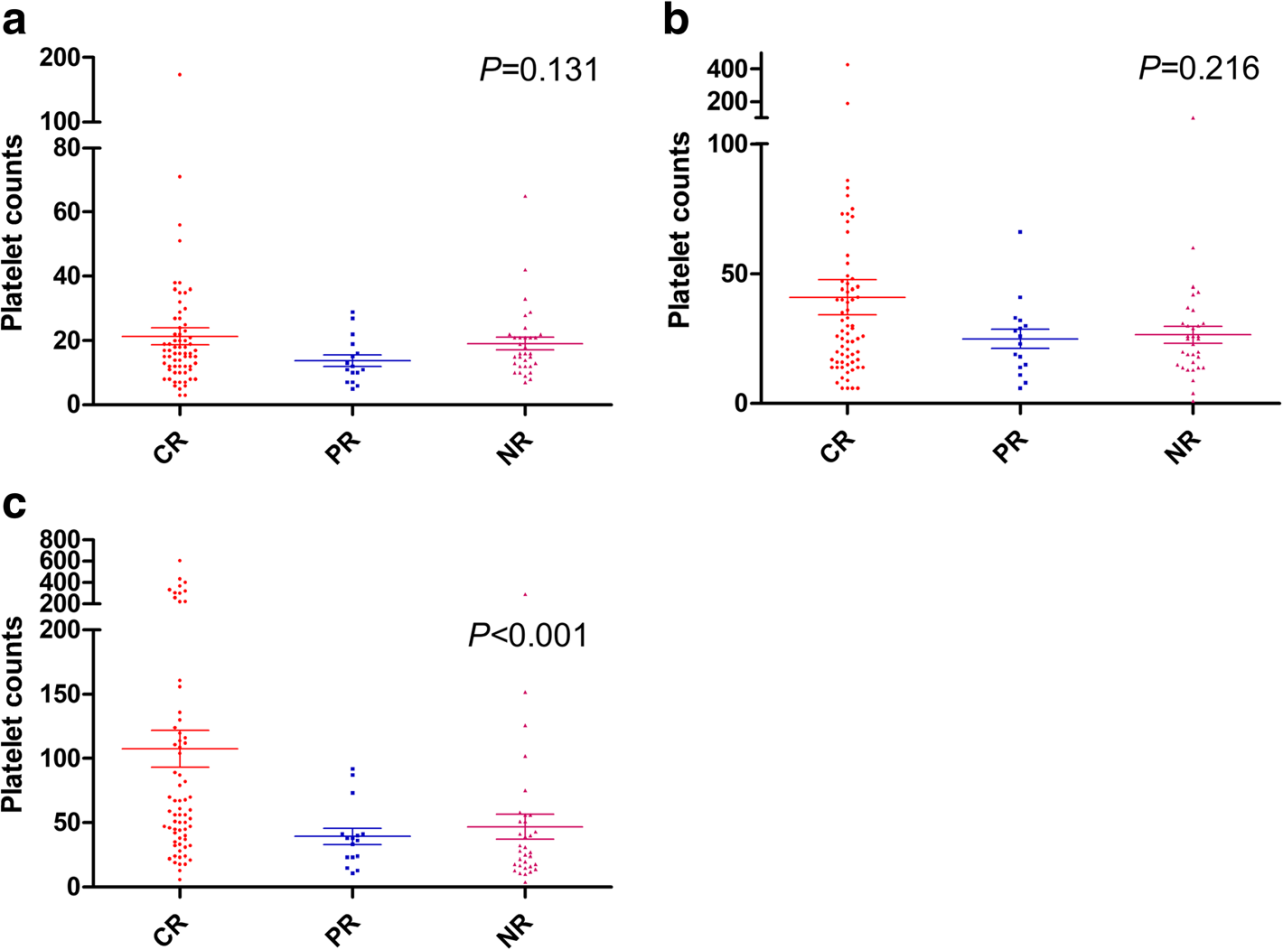
Platelet counts in different therapeutic groups. (**a**) Patients achieving CR (red), PR (blue) or NR (purple)acquired similar median platelet counts on day 7 after D-CAG (**b**) Patients achieving CR, PR or NR acquired similar median platelet counts on day 10 after D-CAG (C) Patients achieving CR acquired higher platelet counts compared with those achieving PR or NR on day 14 after D-CAG

**Fig. 2:**
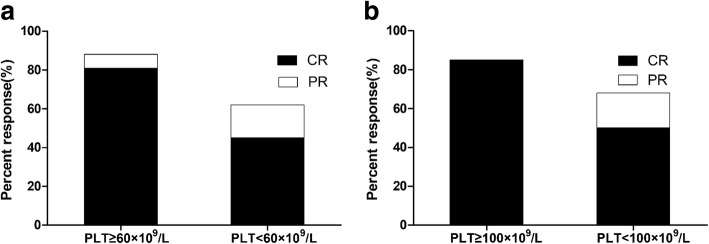
Response rates for selected subgroups. CR was noted in black, and additional patients with partial remission were noted in white. (**a**) Patients with platelet count ≥60 × 10^9^/L acquired significant higher CR and ORR compared with those with platelet count < 60 × 10^9^/L; (**b**) Patients with platelet count ≥100 × 10^9^/L acquired significant higher CR but not ORR compared with those with platelet count < 100 × 10^9^/L

We divided patients into three groups according to the treatment efficacy and analysed peripheral hemogram results, including leukocyte, haemoglobin and platelet counts, on day 7, day 10, and day 14 after the initial therapy. There was a statistically significant difference in terms of platelet count on day 14 in patients with CR compared with those who had PR and NR (*p* < 0.001) (Fig. 1). Median platelet counts in patients with CR, PR and NR were 59.5 × 10^9^/L, 37 × 10^9^/L, and 28 × 10^9^/L, respectively. Therefore, we selected platelet counts threshold of 60 × 10^9^/L (the integer value closest to the median value of the CR group) or 100 × 10^9^/L (normal value) on day 14 after D-CAG and sorted all patients. There was no significant difference in the baseline characteristics between those who achieved and those who did not achieve platelet counts≥60 × 10^9^/L or 100 × 10^9^/L (Table 2). The CR in patients with platelet counts≥60 × 10^9^/L and < 60 × 10^9^/L were 80.9 and 45.3%, respectively (*p* < 0.001). Patients with platelet counts≥100 × 10^9^/L achieved a higher CR rate compared with those who did not (85.2% vs. 50% *p* = 0.001) (Table 3, Fig. 2).

**Table 2:** Patients’ clinical characteristics post-treatment, by recovery of platelet counts

Characteristics	PLT ≥ 60 × 10^9^/L (*N* = 42)		PLT < 60 × 10^9^/L (*N* = 75)		*P*	PLT ≥ 100 × 10^9^/L (*N* = 27)		PLT < 100 × 10^9^/L (*N* = 90)		*P*
	N	Median (range)	N	Median (range)		N	Median (range)	N	Median (range)	
Age(years)		68.5(60–87)		66(60–84)	0.458		69(60–87)		66(60–84)	0.619
Sex					0.706					0.435
Male	22		42			13		51		
Female	20		33			14		39		
ECOG					0.076					0.18
0–2	38		58			25		71		
≥3	4		17			2		19		
Diagnosis					0.305					0.771
M0	0		3			0		3		
M1	9		10			3		16		
M2	22		35			14		43		
M4	2		6			2		6		
M5	5		13			5		13		
M6	2		8			2		8		
Unknown	2		0			1		1		
Primary karyotype					0.202					0.525
Good	0		1			0		1		
Intermediate	36		53			23		66		
Poor	4		14			3		15		
Unavailable	2		7			1		8		
FLT3-ITD mutation					0.81					0.681
Positive	4		9			2		11		
Negative	34		54			22		66		
Missing	4		12			3		13		
NPM1 mutation					0.609					0.722
Positive	10		14			5		19		
Negative	28		50			19		59		
Missing	4		11			3		12		
CEBPα mutation					0.126					0.107
Positive	9		7			7		9		
Negative	29		52			17		64		
Missing	4		16			3		17		
C-Kit mutation					1.000					1.000
Positive	2		2			1		3		
Negative	36		58			23		71		
Missing	4		15			3		16		
Initial CBC										
WBC (×10^9^/L)		11.575 (0.3–310.3)		6.23 (0.7–239.24)	0.475		4.02 (0.3–196.8)		9.29 (0.7–310.3)	0.452
Hb (×g/L)		89.5 (56–128)		81 (47–139)	0.097		89 (57–113)		82 (47–139)	0.785
PLT (×10^9^/L)		56.5 (8–804)		43 (9–575)	0.106		72 (8–804)		44 (9–789)	0.167

*ECOG* Eastern Cooperative Oncology Group, *WBC* white blood cells, *Hb* hemoglobin, *PLT* platelet;

**Table 3:** Platelet counts on day 14 reflect response and survival

	PLT ≥ 60 × 10^9^/L (*N* = 42)	PLT < 60 × 10^9^/L (*N* = 75)	*P*	PLT ≥ 100 × 10^9^/L (*N* = 27)	PLT < 100 × 10^9^/L (*N* = 90)	*P*
ORR (%)	88.1	62.7	0.003	85.2	67.8	0.078
CR (%)	80.9	45.3	< 0.001	85.2	50	0.001
NR (%)	11.9	37.3		14.8	32.2	
OS (months)	16.5	9.1	0.009	31	10.1	0.003
First CR						
DFS (months)	16.3	7.4	0.024	16.9	8.9	0.006

*PLT* platelet, *CR* complete remission, *ORR* overall response rate, *NR* no remission, *OS* overall survival, *DFS* disease-free survival

For patients with intermediate cytogenetics at the time of diagnosis, CR was achieved by 53 patients (59.6%). Ten patients (11.2%) had PR, and twenty-six patients (29.2%) did not respond. For poor cytogenetics, CR was 61.1% (11/18), and PR was 16.7% (3/18). There were no significant differences in the CR and ORR rates between the two different cytogenetic risk groups (X^2^ = 0.015 *p* = 0.902; X^2^ = 0.363 *p* = 0.547).

For patients harbouring FLT3-ITD mutations or wild-type FLT3-ITD, the CR rates were 46.2 and 62.5%, respectively (*p* = 0.261). Patients with wild-type FLT3-ITD achieved higher ORR compared with those with FLT3-ITD mutations (77.3% vs. 46.2% *p* = 0.042). Patients with or without NPM1 mutations had a CR rate of 62.5% and 58.9% (*p* = 0.758), respectively. The ORR was 70.8% and 73.1% (*p* = 0.829).

Multivariate analysis indicated that the platelet counts≥100 × 10^9^/L on day 14 after treatment was associated with CR (Table 4).

**Table 4:** Multivariate analysis for CR and ORR

Characteristics	CR			ORR		
	EXP(B)	*P*	95%CI	EXP(B)	*P*	95%CI
FLT3-ITD mutation		0.716			0.140	
CEBPα mutation		0.159			0.998	
Day-14 PLT	4.231	0.016	1.314–13.622		0.512	

*CR* complete remission, *ORR* overall response rate, *EXP(B)* the exponent of B, *CI* confidence interval, *PLT* platelet

### Survival

Among all patients, one patient underwent bone marrow puncture to evaluate treatment efficacy on day 14 after chemotherapy due to fast recovery of peripheral hemogram. Meanwhile, that day was the last follow-up of this study. Upon final analysis, a total of 77 deaths occurred in this study. The median OS of the cohort group was 13.9 months (range:0–55.1 months) and DFS of the patients achieving CR after the initial chemotherapy was 13.8 months.

### Prognostic factors for OS and DFS

**Fig. 3:**
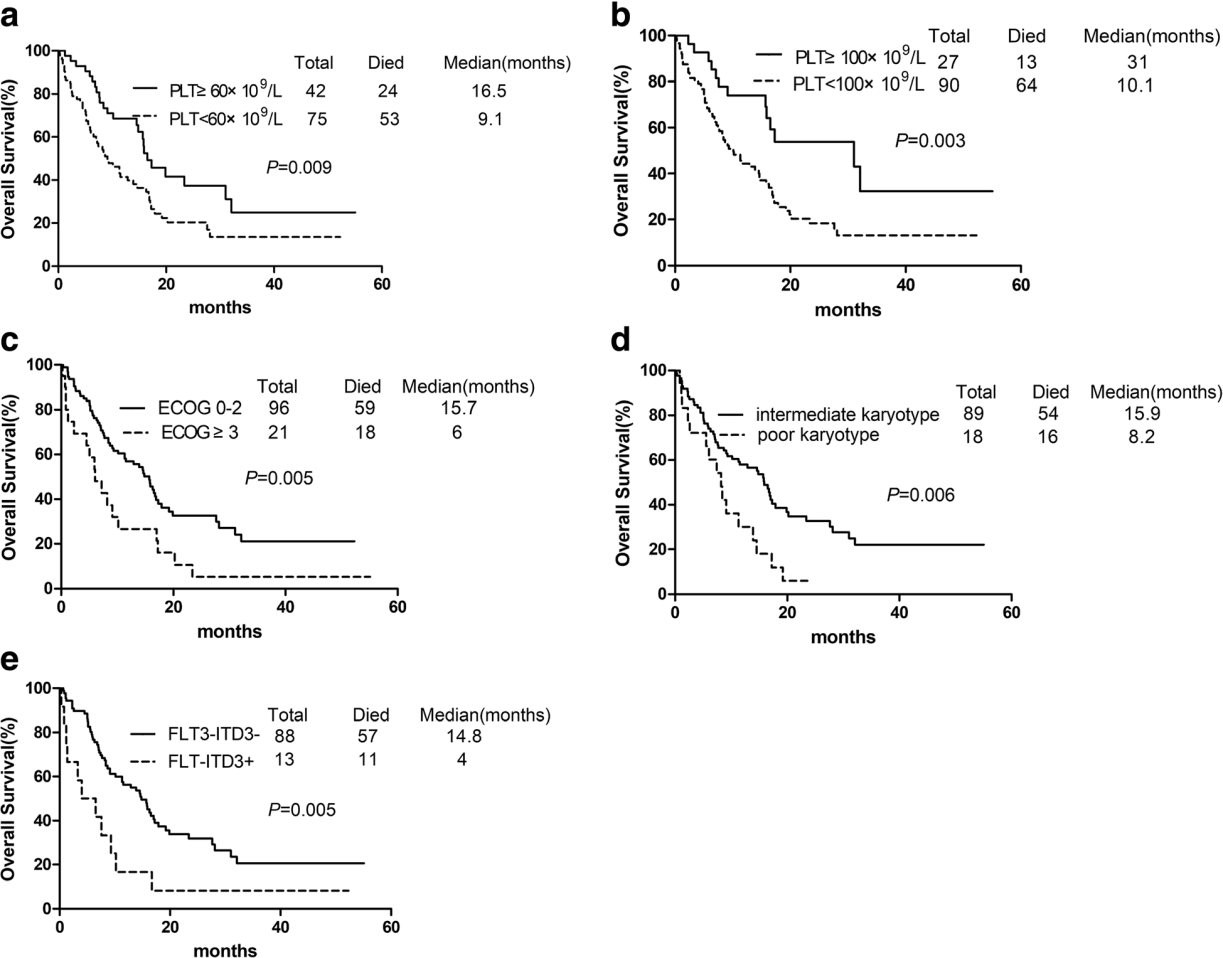
Overall Survival. (**a**) Patients with platelet count ≥60 × 10^9^/L (solid line) had longer OS than PLT < 60 × 10^9^/L (imaginary line) (**b**) Patients with platelet count ≥100 × 10^9^/L (solid line) had longer OS than PLT < 100 × 10^9^/L (imaginary line) (**c**) Patients with good performance status (solid line) had longer OS than poor status (imaginary line) (**d**) Patients with intermediate karyotype (solid line) had longer OS than poor karyotype (imaginary line) (**e**) Patients without FLT-ITD3 mutation (solid line) had longer OS than with mutation (imaginary line)

**Fig. 4:**
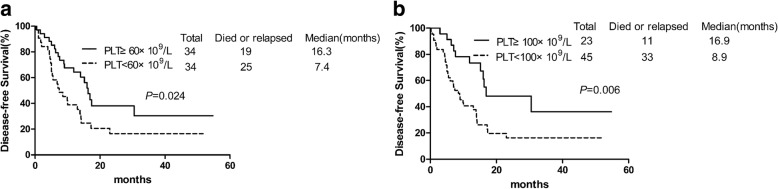
Disease-free Survival. (**a**) Patients with platelet count ≥60 × 10^9^/L (solid line) had longer DFS than PLT < 60 × 10^9^/L (imaginary line) (**b**) Patients with platelet count ≥100 × 10^9^/L (solid line) had longer DFS than PLT < 100 × 10^9^/L (imaginary line)

We dichotomized patients into those achieving a platelet count≥60 × 10^9^/L or 100 × 10^9^/L vs. those who did not. The early recovery of platelets was not associated with the patient age, sex, karyotype or pre-treatment peripheral blood cell counts (Table 2). OS was significantly longer among patients achieving a platelet count≥60 × 10^9^/L or 100 × 10^9^/L compared to patients who did not, after initiation of the first cycle of induction therapy (median OS: 16.5 vs. 9.1 months *p* = 0.009 and 31 vs. 10.1 months *p* = 0.003 Table 3 Fig. 3a, b). As showed in Table 5, patients with ECOG 0–2 had significantly longer OS compared to those with ECOG≥3 (median value: 15.7 vs. 6 months *p* = 0.005 Fig. 3c). Survival was shorter for patients with poor karyotype in comparison with patients with intermediate karyotype (median OS: 8.2 vs. 15.9 months *p* = 0.006 Fig. 3d). Median OS was 14.8 months for patients without FLT-ITD3 mutation and 4 months for patients with mutation (Fig. 3e). CR Patients after first cycle of induction treatment with receiving with platelet counts≥60 × 10^9^/L or 100 × 10^9^/L also acquired longer DFS (median value:16.3 vs. 7.4 months *p* = 0.024 and 16.9 vs. 8.9 months *p* = 0.006 Fig. 4). In an exploratory analysis using a multivariate Cox proportional hazards model, poor cytogenetics (*p* = 0.010) and FLT3-ITD mutation (*p* = 0.007) were identified as the independent adverse prognostic factors, but not platelet count on day 14 (*p* = 0.091). Platelet count on day 14 was of independent prognostic importance for DFS (*p* = 0.035).

**Table 5:** Prognostic factors of overall survival and disease-free survival

Characteristics	OS					DFS				
	Univariate analysis		Multivariate analysis			Univariate analysis		Multivariate analysis		
	Median	*P*	HR	*P*	95%CI	Median	*P*	HR	*P*	95%CI
Age		0.131					0.573			
60–70	15.8					14				
≥70	8.2					10				
Sex		0.858					0.906			
Male	12.9					13.8				
Female	14.6					13				
ECOG		0.005	1.748	0.075	0.945–3.233		0.109			
0–2	15.7					13.8				
≥3	6					5				
FAB classification		0.67					0.451			
M0	4.5					/				
M1	10.2					10				
M2	13.9					13				
M4	8.7					14.1				
M5	11.3					14.2				
M6	15.7					6.3				
Primary karyotype		0.006	2.272	0.010	1.213–4.258		0.045	1.768	0.127	0.850–3.680
Intermediate	15.9					15.2				
Poor	8.2					7				
FLT3-ITD mutation		0.005	2.669	0.007	1.304–5.461		0.352			
Positive	4					7.6				
Negative	14.8					13.8				
NPM1 mutation		0.987					0.782			
Positive	16.5					14.2				
Negative	13.9					10				
CEBPα mutation		0.132					0.089			
Positive	23.4					17.4				
Negative	13.9					10				
C-Kit mutation		0.067					0.987			
Positive	5					16				
Negative	14.6					11.9				
Day-14 platelet count		0.003	0.571	0.091	0.298–1.094		0.006	0.468	0.035	0.231–0.947
PLT ≥ 100 × 10^9^/L	31					16.9				
PLT < 100 × 10^9^/L	10.1					8.9				

*OS* overall survival, *DFS* disease-free survival, *HR* hazard ratio, *CI* confidence interval, *ECOG* Eastern Cooperative Oncology Group, *PLT* platelet

## Discussion

This study targeted elderly AML patients who received decitabine-based chemotherapy. Reliable clinical or molecular parameters that can be used as prognostic marker of benefit from decitabine-based chemotherapy in patients with AML are not defined. An interesting finding from this study was that differences at the platelet level on day 14 after D-CAG correlate with response to decitabine-based chemotherapy and survival benefit in elderly AML patients. Based on a data analysis from 6283 AML patients receiving cytarabine-based therapy, Walter and colleagues [19] found that the patients with CR had superior relapse-free survival (RFS) compared with those achieving CRi (CR with incomplete platelet recovery), suggesting that platelet recovery may be potentially associated with survival. The consensus finding is that a quicker time to platelet recovery or platelet counts≥500 × 10^9^/L at 28 days after one course of induction chemotherapy initiation correlated with RFS and OS in patients with acute leukaemia who entered CR [20, 21].

Nevertheless, it remains controversial which time points after induction are of most prognostic significance or what quantifiable levels of minimal residual disease are most reliable in defining the prognosis of patients [20]. According to the previous study, the platelet count after induction therapy may serve as an index reflecting the amount of minimal residual for AML patients with CR, as well as the degree of recovery upon exposure of normal progenitors to chemotherapy injure [20, 22]. Our observation of close relationships among platelet count, CR and survival seem to be based on a similar phenomenon.

Unlike unaccompanied HMA treatments, which require several courses (four to six cycles) to achieve a response and are often needed to discern the effect of the therapy, responses could be observed after one cycle of conventional chemotherapy, including decitabine-based chemotherapy [4, 20, 21, 23]. It has been reported that low-dose decitabine promotes normal megakaryocyte outgrowth and differentiation of normal megakaryocytes into platelets [12, 13]. To our knowledge, the impact of early platelet regeneration to prognosis after decitabine-based chemotherapy has not been studied in a systematic fashion in AML before.

We evaluated the effect of baseline biomarkers, molecular mutation or clinical variables, especially the regeneration of haematopoiesis, on day 7, day 10 and day 14 after D-CAG. Consistent with the results from our previous study [4], there was no statistical significance of CR rate between patients with poor and intermediate karyotype. Also, patients with or without FLT3-ITD mutation acquired similar CR rate. Those results indicated that patients at high risks could also benefit from decitabine induction therapy. The association between molecular or clinical biomarkers and decitabine response may be confounded by the variations in enzymes responsible for the activation and metabolism of decitabine [9]. Thus, it is clear that the evaluation of data collected after treatment may be more useful compared with evaluation of pre-treatment data, including cytogenetics and various genetic abnormalities [22].

With the aim of identifying patients who will derive the greatest clinical benefit from decitabine-based chemotherapy, we analysed post-treatment findings to assess CR and OS that have focused on laboratory parameters, measured during the course of induction or at the time of CR. Here, we analysed whether platelet count, a much simpler test, on days 7, 10, and 14 after induction therapy might also be associated with CR and OS. We found superior CR and survival in elderly AML patients with early recovery of platelet counts after one course of D-CAG and demonstrated that early hyper-recovery of platelet counts is a prognostic factor independent of other known prognostic factors in elderly AML patients who received D-CAG. Our results revealed that patients achieving CR, PR or CR had similar peripheral hemogram results on day 7 and day 10 after D-CAG. On day 14, patients achieving CR attained higher platelet counts compared with those achieving PR or NR (59.5 × 10^9^/L, 37 × 10^9^/L and 28 × 10^9^/L *p* < 0.001). CR was shown to be an important prognostic marker of longer survival [24]. Furthermore, we dichotomized patients into those who achieved a platelet count≥60 × 10^9^/L (the integer value closest to the median count of the CR group) or ≥ 100 × 10^9^/L (normal value) on day 14 after D-CAG versus those who did not. Using this category, we also found that differences at the platelet level correlated with response to decitabine-based chemotherapy and superior survival in elderly AML patients.

## Conclusions

Poor ECOG, unfavourable cytogenetics and FLT3-ITD mutation are, prior to treatment, poor prognostic indicators for elderly patients with AML. Based on the results of this study, a platelet count≥60 × 10^9^/L or ≥ 100 × 10^9^/L on day 14 after decitabine combined chemotherapy was associated with increase response rate in the clinic. Also, the association of platelet count with longer OS and DFS was revealed through univariate analyses. With better definitions of subsets of patients, there is likely to be a role for aiding the physician decision-making process in our various prognostic models. However, since this study is an analysis of a limited sample size, independent validation of large-scale patients is needed in the future.

## Additional file

Additional file 1: **Figure S1.** Enrollment and outcomes. 127 AML patients were administrated with decitabine for 5 consecutive days (day 1–5) and G-CSF (day 0–9) in combination with cytarabine (day 3–9), aclarubicin for 4 days (day 3–6) (D-CAG). Only 117 patients were available to obtain blood routine and efficacy data after the first course of treatment. The results showed 68 patients acquired CR, 16 patients PR and 33 patients NR. The platelet counts on day 14 after D-CAG of three groups were 59.5 × 10^9^/L, 37 × 10^9^/L and 28 × 10^9^/L. Partial patients who acquired PR or NR received second cycle of D-CAG induction therapy. (DOC 52 kb)

## Abbreviations

AML: Acute myeloid leukaemia
CI: Confidence interval
CR: Complete remission
DFS: Disease-free survival
Hb: Haemoglobin
HR: Hazard ratios
ORR: Overall response rate
OS: Overall survival
PR: Partial remission
RFS: Relapse-free survival
WBC: White blood cells
