# Supplementary material for: Early recovery of the platelet count after decitabine-based induction chemotherapy is a prognostic marker of superior response in elderly patients with newly diagnosed acute myeloid leukaemia
Source: BMC Cancer. 2018 Dec 19;18:1269. doi: 10.1186/s12885-018-5160-5 (PMC6299938; doi:10.1186/s12885-018-5160-5)
Supplement: Supplementary file 1 — Figure S1. Enrollment and outcomes. 127 AML patients were administrated with decitabine for 5 consecutive days (day 1–5) and G-CSF (day 0–9) in combination with cytarabine (day 3–9), aclarubicin for 4 days (day 3–6) (D-CAG). Only 117 patients were available to obtain blood routine and efficacy data after the first course of treatment. The results showed 68 patients acquired CR, 16 patients PR and 33 patients NR. The platelet counts on day 14 after D-CAG of three groups were 59.5 × 109/L, 37 × 109/L and 28 × 109/L. Partial patients who acquired PR or NR received second cycle of D-CAG induction therapy. (DOC 52 kb) [file 12885_2018_5160_MOESM1_ESM.doc]

127 AML patients from September 2011 to April 2016 receiving DCAG induction chemotherapy

4 patients died

6 patients withdraw after treatment

117 patients were estimated by response rate and blood routine values

the first induction therapy

68 patients achieved CR

16 patients achieved PR

33 patients achieved NR

second cycle(partial patients）

CR:7 patients

Median day-14 PLT 59.5×10^9^/L

Median day-14 PLT counts 37×10^9^/L

Median day-14 PLT counts 2×10^9^/L
